# Changes in parotid gland morphology and function in patients treated with intensity-modulated radiotherapy for nasopharyngeal and oropharyngeal tumors

**DOI:** 10.1007/s11282-013-0151-3

**Published:** 2013-08-25

**Authors:** Kenichi Obinata, Motoyasu Nakamura, Marco Carrozzo, Iain Macleod, Andrew Carr, Shinichi Shirai, Hitoshi Ito

**Affiliations:** 1 Department of Dental Radiology, Center for Dental Clinics, Hokkaido University Hospital, Kita-13, Nishi-7, Kita-ku, Sapporo, Hokkaido 060-8586 Japan; 2 Nakamura Dental Clinic, Sapporo, Japan; 3 Department of Oral Medicine, University of Newcastle upon Tyne, Newcastle upon Tyne, UK; 4 Department of Dental Radiology, University of Newcastle upon Tyne, Newcastle upon Tyne, UK; 5 Omni Dentix, Sapporo, Japan

**Keywords:** Intensity-modulated radiotherapy (IMRT), Parotid gland, Nasopharyngeal and oropharyngeal tumors, Morphological and functional changes

## Abstract

**Objective:**

To evaluate the morphological changes of the parotid glands in patients treated with intensity-modulated radiotherapy (IMRT) for nasopharyngeal and oropharyngeal tumors and the correlations with parotid function.

**Methods:**

Ten patients with nasopharyngeal and oropharyngeal tumors treated with IMRT between May 2009 and January 2010 at Hokkaido University Hospital were included in this study. In the morphological assessment of the parotid glands, the sizes and computed tomography (CT) numbers of the bilateral parotid glands before and after IMRT with CT were calculated. For functional assessment of the parotid glands, we conducted the Saxon test and used a visual analog scale (VAS) for xerostomia evaluation.

**Results:**

Reductions in saliva secretion were observed in the patients treated with IMRT for nasopharyngeal and oropharyngeal tumors, and there was a significant correlation between the reduction in saliva secretion and the VAS. The reductions in the parotid gland size and CT number were larger on the ipsilateral side than on the contralateral side. The reduction in saliva secretion was not significantly correlated with the reduction in parotid gland size, but was significantly correlated with the reduction in CT number.

**Conclusions:**

Morphological and functional changes of the parotid glands were observed after IMRT for nasopharyngeal and oropharyngeal tumors, and preservation of the contralateral parotid glands was only partly achieved. Among the morphological changes of the parotid glands, the CT number may be considered a predictor of parotid function after radiotherapy.

## Introduction

Radiotherapy plays an important role in head and neck tumor treatment because of the cosmetic and functional preservation that becomes possible. Despite advances in radiotherapy equipment and irradiation technology, morbidity related to radiotherapy is not completely avoidable [1]. Xerostomia is a common adverse effect of radiotherapy for head and neck tumors, and results in reduced patient quality of life by inducing dysphagia, dysgeusia, caries, and periodontitis [2]. In recent years, our hospital has used intensity-modulated radiotherapy (IMRT) for patients with head and neck tumors to enable preservation of the major salivary glands. It has been reported that IMRT is superior to conventional radiotherapy in regard to dose, dosimetry, and preservation of salivary gland functions [3, 4]. However, we have experienced a variety of subjective complaints of xerostomia in patients treated with IMRT. The present study aimed to evaluate the morphological changes of the parotid glands in patients treated with IMRT for nasopharyngeal and oropharyngeal tumors and to assess the correlations with the functioning of the parotid glands.

## Patients and methods

### Patient characteristics

Between April 2009 and January 2010, six patients with nasopharyngeal tumors and four patients with oropharyngeal tumors were treated with IMRT at the Division of Radiotherapy, Hokkaido University Hospital. Written informed consent for IMRT was obtained from all patients. Histologically, one patient with tonsil lesions was diagnosed with non-Hodgkin’s lymphoma, and six patients with nasopharyngeal tumors and three patients with oropharyngeal tumors were diagnosed with squamous cell carcinomas. The patients were six males and four females (mean age 64 years, age range 40–85 years). Clinically, the tumors were diagnosed as T1 in two patients, T2 in four, and T3 in four, and the nodal involvement was N0 in three patients, N1 in one, N2a in two, and N2b in four (Table 1). Seven of the ten patients received concurrent chemotherapy consisting of CDDP (cisplatin) (six patients) and TPF (taxotere + cisplatin + 5-fluorouracil) (one patient).

**Table 1:** Patient and tumor characteristics

Patient no.	Age/sex	Primary site	TNM	Pathology	Chemotherapy
1	59/F	NP (p-lt wall)	T2N2bM0	SCC	TPF
2	68/M	OP (rt tonsil)	T3N0M0	SCC	CDDP
3	73/M	OP (rt tonsil)	T3N2bM0	ML	
4	45/F	NP (rt wall)	T2N2M0	SCC	CDDP
5	66/F	NP (rt wall)	T1N0M0	SCC	CDDP
6	71/F	OP (rt tonsil)	T2N2bM0	SCC	CDDP
7	40/M	NP (rt wall)	T3N1M0	SCC	CDDP
8	77/M	NP (lt wall)	T1N2M0	SCC	
9	56/M	OP (lt tonsil)	T2N2bM0	SCC	CDDP
10	85/M	NP (u-p wall)	T3N0M0	SCC	

*NP* nasopharynx, *OP* oropharynx, *p* posterior, *u* upper, *rt* right, *lt* left, *SCC* squamous cell carcinoma, *ML* malignant lymphoma; *TPF* taxotere + cisplatin + 5-fluorouracil, *CDDP* cisplatin

### Parotid sizes and computed tomography (CT) numbers

**Fig. 1:**
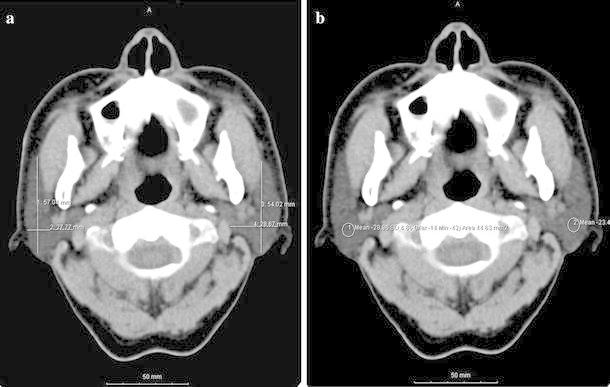
**a** Method for determining the sizes of the parotid glands. Cross-sectional long/short diameters, showing the maximum area of the parotid glands, were measured on axial plain CT images. **b** Method for determining the CT numbers of the parotid glands. *Circular regions* of interest were selected on the posterolateral regions of the parotid glands, avoiding the retro-mandibular veins

The sizes and CT numbers of the bilateral parotid glands were determined on axial plain CT images before and after IMRT. Cross-sectional long/short diameters, showing the maximum area of the parotid glands, were also measured. The axial scan that showed the fewest artifacts caused by metal restorations and crowns in the oral cavity and had the largest size was selected to calculate the CT numbers of the parotid glands. The posterolateral regions of the parotid glands avoiding the retro-mandibular veins were selected to calculate the CT numbers. The CT numbers were determined three times, and the mean CT number was used as the representative CT number for a parotid gland (Fig. 1a, b).

### Saxon test and visual analog scale (VAS)

As an objective assessment of salivation, we conducted the Saxon test before and after IMRT. The Saxon test was performed in the morning at our outpatient clinic. Patients were instructed not to drink, eat, or smoke for 2 h before the examination. In the Saxon test, saliva production was measured by weighing gauze before and after 2 min of chewing without swallowing. As a subjective assessment of salivation, we applied a VAS for xerostomia. The VAS was a horizontal 100-mm line with vertical anchoring lines. The description used at the left end (0 mm) was “No feeling of dry mouth” and that at the right end (100 mm) was “Feeling dry mouth as severe as it can be”. The CT examination, Saxon test, and VAS were performed within 1 week before the start of IMRT and after the completion of IMRT in each patient.

### Statistical analysis

The changes in the bilateral parotid sizes and CT numbers before and after IMRT were compared with the Saxon test and VAS. Differences between the parotid glands on the ipsilateral side and the contralateral side were compared with a Student’s *t*-test. The relationship between the parotid morphological and functional changes was evaluated with a Spearman rank correlation test. Statistical analyses were carried out using StatView Version 5 (SPSS, Cary, NC). Values of *p* < 0.05 were considered to indicate statistical significance.

## Results

**Fig. 2:**
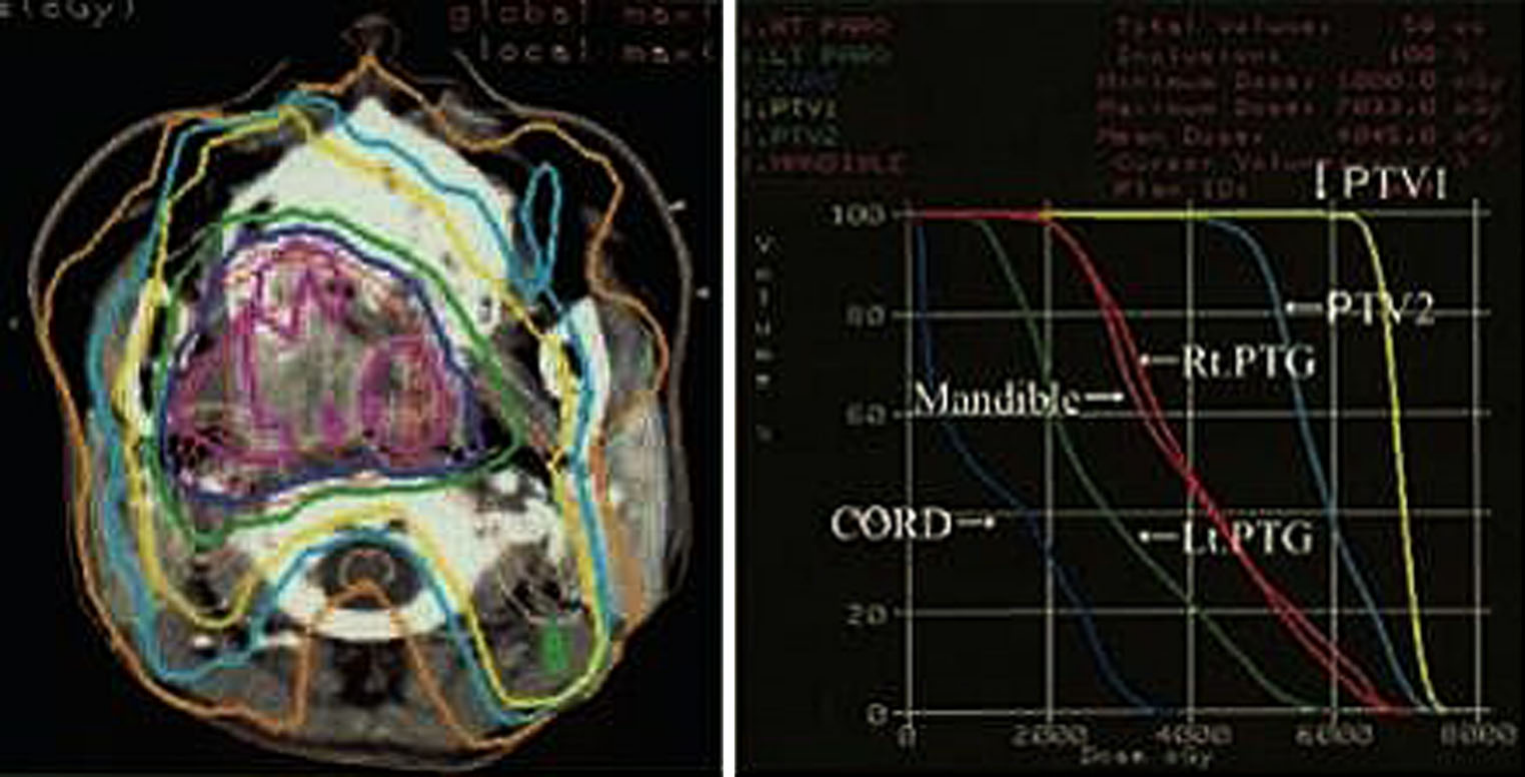
Dose distribution and dose-volume histogram for a patient with an oropharyngeal tumor

**Fig. 3:**
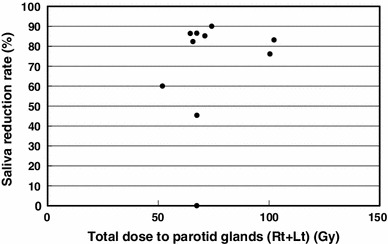
Relationship between the total dose to the bilateral parotid glands and the reduction in salivation. The Spearman rank correlation coefficient is 0.26

**Fig. 4:**
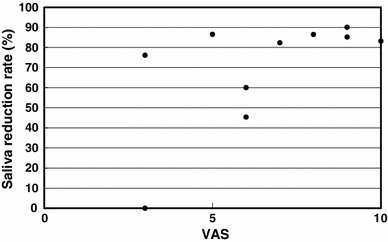
Relationship between the reduction in salivation and the VAS. The Spearman rank correlation coefficient is 0.64 (*p* < 0.05)

**Fig. 5:**
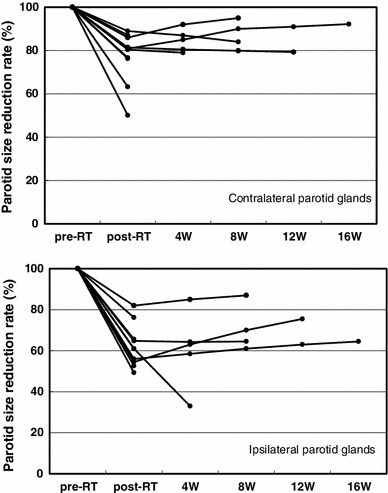
Comparison of the rate of the size reduction between the ipsilateral and contralateral parotid glands. The difference is significant (*p* < 0.02, Student’s *t*-test). *RT* radiotherapy, *W* week

**Fig. 6:**
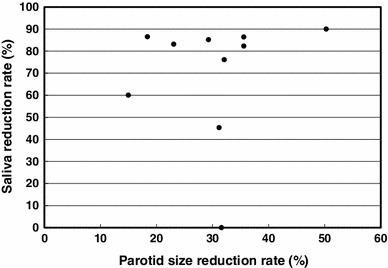
Relationship between the reduction in the parotid size and salivation. The Spearman rank correlation coefficient is 0.32

**Fig. 7:**
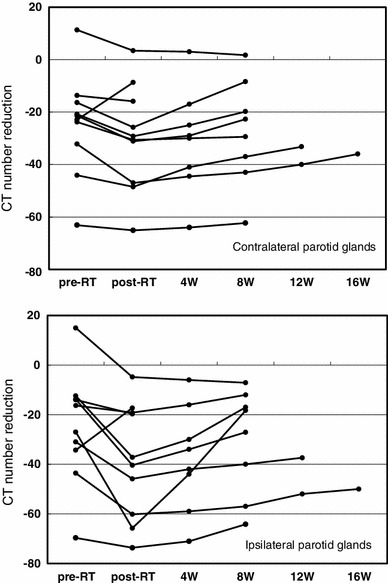
Comparison of the reduction in the CT numbers between the ipsilateral and contralateral parotid glands. The difference is significant (*p* < 0.05, Student’s *t*-test). *RT* radiotherapy, *W* week

**Fig. 8:**
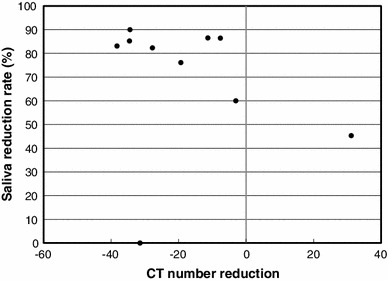
Relationship between the reduction in the CT numbers and salivation. The Spearman rank correlation coefficient is 0.84 (*p* < 0.01)

The total dose to planning target volume (PTV) was 70 Gy in 35 fractions for squamous cell carcinomas and 50 Gy in 25 fractions for malignant lymphomas. The ipsilateral parotid glands were partially included in the PTV (Fig. 2). The mean dose was 41.5 Gy (33.9–68.4 Gy) for the ipsilateral parotid glands and 31.7 Gy (26.0–43.5 Gy) for the contralateral parotid glands. The average saliva secretion was 6.1 g (0.7–21.7 g) before IMRT and 1.4 g (0.3–4.1 g) after IMRT, giving a reduction rate of 69.5 % (0–90.0 %). For the subjective assessment of xerostomia, the VAS varied depending on the patients, and the mean score was 6.6 (2–10) (Table 2). There was no correlation between the total dose to the bilateral parotid glands and the reduction in salivation (*r* = 0.26) (Fig. 3). There was a significant correlation between the reduction in salivation and the VAS (*r* = 0.64, *p* < 0.05) (Fig. 4). The rate of reduction in the parotid gland size was 39.4 % (23.8–50.7 %) for the ipsilateral side and 26.5 % (12.9–44.9) for the contralateral side, and the difference was significant (*p* < 0.02) (Fig. 5). No significant correlation was established between the bilateral reduction in the parotid size and salivation (*r* = 0.32) (Fig. 6). The CT number was reduced by 13.8 (−28.8 to +17.0) for the ipsilateral side and 5.4 (−14.8 to +14.3) for the contralateral side, and the difference was significant (*p* < 0.05) (Fig. 7). There was a significant strong correlation between the reductions in the CT number and salivation (*r* = 0.84, *p* < 0.01) (Fig. 8). It was observed that the CT numbers of the parotid glands tend to revert after completion of IMRT (Figs. 5, 7).

**Table 2:** Doses to PTV and bilateral parotid glands, saliva secretion, and VAS

Patients	Total dose (Gy/fractions)	Parotid mean dose (Gy) (ipsi/cont)	Pre-RT (g/2 min)	Post-RT (g/2 min)	Reduction rate (%)	VAS
1	70/35	39.1/28.3	0.7	0.7	0	3
2	70/35	38.5/29.0	7.5	4.1	45.3	6
3*	50/25	38.0/26.5	4.4	0.6	86.4	8
4	70/35	58.8/43.5	8.9	1.5	83.1	10
5	70/35	38.7/35.5	5.0	0.5	90.0	9
6#	70/35	68.4/32.1	4.6	1.1	76.1	3
7	70/35	37.6/33.5	4.1	0.6	85.2	9
8	70/35	33.9/33.5	3.7	0.5	86.5	5
9	70/35	36.4/29.3	21.7	3.9	82.3	7
10	70/35	26.0/25.9	0.9	0.3	60.0	6
Mean		41.5/31.7	6.1	1.4	69.5	6.6

*ipsi* Ipsilateral, *cont* contralateral, *RT* radiotherapy, *VAS* visual analog scale

* A case of malignant lymphoma

^#^Lymph node metastasis in the ipsilateral parotid gland

## Discussion

IMRT is a newly introduced technique that aims to spare radiosensitive organs while delivering adequate doses to tumors. Initially, prostate cancer was treated with IMRT to prevent adverse effects on the rectum [5]. Many patients treated with radiotherapy for head and neck malignancies suffer from xerostomia, which causes reductions of patient quality of life, including difficulty of speaking and swallowing, as well as taste loss and oral infections [2]. There have been several attempts to reduce radiation damage to the major salivary glands in radiotherapy [3, 4]. In this study, IMRT was applied to preserve the functions of the salivary glands. In most IMRT studies, much effort goes into reducing the parotid gland dose. A number of papers have reported theoretical values for acceptable IMRT doses in the reduction of parotid gland doses [6]. However, only a few reports have dealt with measurements to assess the effects of reducing the gland doses. Marks et al. [7] observed a marked reduction in salivary flows in glands receiving more than 30–40 Gy. Leslie and Dische [8] found that the average parotid salivary flow was 60–70 % of the baseline following 10–14 Gy and undetectable in glands receiving 40–42 Gy [8]. They concluded that the dose required for an optimum effect lies between 15 and 40 Gy. In the present study, we used the Saxon test to measure the saliva output, because it reflects the function of the parotid glands as stimulated salivation rather than unstimulated salivation, which is predominantly from the submandibular glands. The mean dose to the parotid gland for the ipsilateral and contralateral parotid glands was 41.5 Gy and 31.7 Gy, with size reductions of 39.4 and 26.5 %, respectively (*p* < 0.02). Although the saliva flow from each parotid gland was not separately determined, this result may still reflect a dose-dependent morbidity of the parotid gland function, and the threshold beyond which damage becomes prominent may lie around 30 Gy. The saliva reduction rate in this study was 69.5 %, which is comparable to other studies. Although feelings of mouth dryness are very subjective and affected by variability, the present measurements showed significant correlations between the objective saliva output and the Saxon test and VAS. These results indicate that the VAS, reflecting the subjective patient sensation of mouth dryness, is a useful and concise parameter to assess xerostomia after radiotherapy. Teshima et al. [9] evaluated the correlation between parotid volume and saliva production in patients treated with preoperative radiotherapy for oral cancers. They reported that after 30 Gy of irradiation, the mean saliva production decreased from 4.2 to 1.0 g and the post-radiotherapy/pre-radiotherapy parotid volume ratio ranged from 54 to 85 % (mean 71 %) [9]. They also indicated that the post-radiotherapy/pre-radiotherapy parotid volume ratio was inversely and significantly correlated with the amount of post-radiotherapy saliva reduction [9]. The reduction in the parotid gland size in the present study was 39.4 % for the ipsilateral side and 26.5 % for the contralateral side. These findings may demonstrate that IMRT achieved the aim of alleviating the adverse effects on the contralateral parotid glands, taking into consideration the fact that almost all patients in this study were administered 70 Gy of irradiation and that the difficulties in IMRT planning because of the reduced laterality resulted from the tumor staging and occurrence site. Although there was no significant correlation between the reduction in the parotid gland sizes and saliva production, this is likely to arise from the small sample size in this study. There have been no other reports of CT numbers of salivary glands after radiotherapy. Heo et al. [10] reported a quantitative analysis of normal major salivary glands using CT, and reported that the CT numbers varied widely with sex, age, and obesity state (body mass index). Similar to the previous reports, the CT numbers of the patients in the present study varied from −69.7 to +15.0. A reduction in the CT number was observed in 9 of the 10 patients in this study, and the range of the reduction was significantly larger in the ipsilateral parotid gland than in the contralateral parotid gland (*p* < 0.05). Numerous investigators have described the nature of salivary gland damage after irradiation in animal studies [10–13]. The irradiated parotid glands are characterized by significant fibrosis, acinar atrophy, and parenchymal loss [10, 11]. It is known that radiation-induced fatty replacement of bone marrow is the most common osseous abnormality observed in patients with doses as low as 800 cGy [14]. Furthermore, Tartaglino et al. [15] reported that muscle and salivary gland atrophy with fatty replacement may arise from radiation-induced changes in the head and neck. Sagowski et al. [13] reported that progressive vacuolization (30 Gy) developed into lipomatosis (46 Gy) in rat parotid glands. The reductions in the CT numbers after radiotherapy in the present study may have resulted from acinar atrophy, parenchymal loss, and fatty replacement, which would be in agreement with the previous reports. On the contrary, another report demonstrated increased attenuation and enhancement as well as atrophy of salivary glands after radiotherapy evaluated by contrast-enhanced CT [14]. The enhancements of the salivary glands may be caused by contrast material leakage owing to increased vascular permeability or an increase in the extracellular space owing to a diminished number of acini [14]. Although the CT scans obtained in the present study were plain (non-enhanced), one patient showed an increase in the CT number. This patient complained of parotid tenderness after the radiotherapy, and may have been affected by sialadenitis. This could be a result of increases in water content caused by edema of the inflamed parotid gland. Nomayr et al. [16] reported that after irradiation of 70 Gy, salivary gland edema, corresponding to sialadenitis, was detected in 2 of 9 cases (parotid gland) and 4 of 13 cases (submandibular gland). The present study may provide additional insight into the effects of radiation-induced salivary gland injury. Among the morphological changes of the parotid glands, the CT number can also be a predictor of the function of saliva production after radiotherapy. Further studies of this matter are necessary, including attention to the morphological changes of submandibular/sublingual glands with longer follow-up periods and comparisons of IMRT with conventional RT.
